# Performance of a Newly Isolated Salt-Tolerant Yeast Strain *Sterigmatomyces halophilus* SSA-1575 for Azo Dye Decolorization and Detoxification

**DOI:** 10.3389/fmicb.2020.01163

**Published:** 2020-06-11

**Authors:** Rania Al-Tohamy, El-Refaie Kenawy, Jianzhong Sun, Sameh Samir Ali

**Affiliations:** ^1^Biofuels Institute, School of the Environment and Safety Engineering, Jiangsu University, Zhenjiang, China; ^2^Polymer Research Group, Department of Chemistry, Faculty of Science, Tanta University, Tanta, Egypt; ^3^Botany Department, Faculty of Science, Tanta University, Tanta, Egypt

**Keywords:** azo dyes, bioremediation, halotolerant yeasts, lignin-modifying enzymes, *Sterigmatomyces halophilus*, detoxification, microtox assay, wood-feeding termites

## Abstract

The effective degradation of hazardous contaminants remains an intractable challenge in wastewater processing, especially for the high concentration of salty azo dye wastewater. However, some unique yeast symbionts identified from the termite gut system present an impressive function to deconstruct some aromatic compounds, which imply that they may be valued to work on the dye degradation for various textile effluents. In this investigation, a newly isolated and unique yeast strain, *Sterigmatomyces halophilus* SSA-1575, was identified from the gut system of a wood-feeding termite (WFT), *Reticulitermes chinensis*. Under the optimized ambient conditions, the yeast strain SSA-1575 showed a complete decolorization efficiency on Reactive Black 5 (RB5) within 24 h, where this azo dye solution had a concentration of a 50 mg/L RB5. NADH-dichlorophenol indophenol (NADH-DCIP) reductase and lignin peroxidase (LiP) were determined as the key reductase and oxidase of *S. halophilus* SSA-1575. Enhanced decolorization was recorded when the medium was supplemented with carbon and energy sources, including glucose, ammonium sulfate, and yeast extract. To understand a possible degradation pathway well, UV-Vis spectroscopy, FTIR and Mass Spectrometry analyses were employed to analyze the possible decolorization pathway by SSA-1575. Determination of relatively high NADH-DCIP reductase suggested that the asymmetric cleavage of RB5 azo bond was mainly catalyzed by NADH-DCIP reductase, and finally resulting in the formation of colorless aromatic amines devoid of any chromophores. The ecotoxicology assessment of RB5 after a decolorization processing by SSA-1575, was finally conducted to evaluate the safety of its metabolic intermediates from RB5. The results of Microtox assay indicate a capability of *S. halophilus* SSA-1575, in the detoxification of the toxic RB5 pollutant. This study revealed the effectiveness of halotolerant yeasts in the eco-friendly remediation of hazardous pollutants and dye wastewater processing for the textile industry.

## Introduction

Undoubtedly, the rapid development of industrialization is always associated with an increase in the complexity and waste toxicities, which are causing a cost, which is paid in terms of environmental pollution (Ali et al., 2018, 2020a,b). It is approximated that 10,000 different dyes, with an estimated annual production of 280,000 tons, are commercially available worldwide; and azo dyes represent over 60% of the total dyes (Patel et al., 2017; Pattanaik et al., 2020). It is estimated that 20–50% of these dyes remain unfixed during the dyeing processes and ultimately end up in the dye effluents (Giovanella et al., 2020), leading to severe pollution of water supplies in the vicinity of dyeing industries (Neetha et al., 2019). Hence, many governments have established environmental laws and restrictions not only for aesthetic reasons but also due to the serious ecological risks and toxicity on aquatic flora, as well as the mutagenicity and carcinogenicity of azo dye degradation products (Yang et al., 2018; Ali et al., 2019; Tkaczyk et al., 2020).

Azo dyes, which represent one of the largest consuming dyestuff categories around the world, are major contributors that trigger severe environmental pollution issues due to their extensive use in textile and leather dyeing, paper printing, cosmetics and many other industries (Pattanaik et al., 2020; Tkaczyk et al., 2020). Azo dyes are often recalcitrant to biodegradation processes, as their chemical structure contains one or more azo bonds (Arora, 2015). Effluents with a residual azo dye concentration of 10–200 mg/L, are highly colored and aesthetically unpleasant (Martorell et al., 2017). Consequently, the disposal of azo dyes from their effluents, is of utmost importance before being discharged into an environment.

There are several physico-chemical techniques for the disposal of azo dyes from effluents, including adsorption, membrane filtration, flocculation, photocatalysis, coagulation, and ozone (Lucas et al., 2006; Dafale et al., 2008). However, high toxicity by-products, high treatment costs, time-consuming, labor-intensive and low efficiency are the main drawbacks of using these conventional techniques (Chen et al., 2015; Martorell et al., 2017). Conversely, biological approaches, including microorganisms and/or their lignin-modifying enzymes (LMEs) have received greater attention in recent years as a way of treating azo dyeing effluents. The biodegradation techniques to remove azo dyes from effluents present the following advantages: optimal dye disposal efficiency, high performance at low concentrations as well as being an environmental eco-friendly alternative, leading to the formation of non-toxic residues at low operating costs (Oturkar et al., 2011; Alegbeleye et al., 2017). Over the past decades, microbial degradation of azo dyes has been accomplished by bacteria, actinomycetes, yeasts, fungi, and algae (Enayatizamir et al., 2011; Agrawal et al., 2014; Zhang et al., 2015; Ali et al., 2019; Giovanella et al., 2020). On the other hand, some extracellular microbial enzymes have been reported for efficient degradation of recalcitrant azo dyes, including laccase (Lac), lignin peroxidase (LiP), manganese dependent peroxidase (MnP), and NADH-dichlorophenol indophenol (NADH-DCIP) reductase (Guo et al., 2019; Giovanella et al., 2020). The LMEs that possess a remarkable capability to degrade lignin and lignin-like substances mainly include LiP, MnP, and Lac along with other supporting enzymes, such as aryl alcohol oxidase, glyoxal oxidase, oxalate decarboxylase and versatile peroxidase (Iqbal et al., 2011). Nowadays, the immobilized LMEs are an innovative biological approach for the degradation, decolorization or detoxification of dye-based wastewater effluents (Oturkar et al., 2011). The immobilized LEMs have various advantages, such as high enzyme/substrate ratio, enhanced level of product stability, improved hyperactivity, less chance of contamination, increased functional efficacy, and an enhanced level of continuous operation (Bilal et al., 2017).

The effluents of the wastewater textile industry contain azo dyes and a high concentration of salts. It has been reported that the presence of salt induces the high osmotic pressure, which challenges microorganisms in the processing of textile wastewater (Khalid et al., 2012; Liu et al., 2017; Yang et al., 2018; Guo et al., 2019). Halophilic and halotolerant (salt-tolerant) microorganisms have biological advantages and provide high treatment efficiency, especially for the high concentration of salty azo dye wastewater (Yu et al., 2015; Guo et al., 2019; Giovanella et al., 2020). Halophiles are a unique group of organisms that live in high saline environments. These organisms require the salinity to survive. On the other hand, halotolerant organisms can grow under saline conditions, but the elevated concentrations of salt are not necessary for their growth (Kavynifard et al., 2016). Dyeing wastewater always contains a high salt concentration between 3 and 10% NaCl (Guo et al., 2020). Therefore, it is important to use microbial strains capable of tolerating high salt concentrations >3% during the treatment of textile effluents (Giovanella et al., 2020). As they can survive in hypersaline conditions, halotolerant and halophilic microorganisms are the best alternatives to decolorize azo dyes in wastewater effluents with a high load of salts.

The bioremediation of extreme habitats requires microorganisms that are adapted to such environments. As far as it is known, many bacteria and filamentous fungi were reported for their dye decolorization efficiency. However, relatively limited studies investigated the biodegradation of azo dyes by halotolerant unicellular fungi, such as yeasts (Song et al., 2017; Guo et al., 2019). Hence, it is significant to search for more halotolerant yeasts valued for the treatment of azo dye wastewater with high salinity. Recently, some strains of *Pichia occidentalis, Sterigmatomyces halophilus*, and *Scheffersomyces spartinae* were identified (Tan et al., 2016; Ali et al., 2017; Song et al., 2017). These strains exhibited the capability to tolerate various extreme conditions such as high salts. However, to the best of the authors' knowledge, to date, there have been no reports on the decolorization and detoxification of azo dyes by yeast strains belonging to *S. halophilus*. Water activity (*a*_w_) is one of the major environmental factors affecting yeast growth (Tokuoka, 1993). Compared with salt-tolerant ascomycetous yeasts, the water relations of basidiomycetous yeast genera such as *Sterigmatomyces* has been poorly investigated, maybe due to the fact that this group is generally not involved in food spoilage or industrial applications (Tekolo et al., 2010). However, several strains of *S. halophilus* have been reported on, for their medical potential. For example, *S. halophilus* strain N16 has been considered a novel fish immunostimulant against vibriosis diseases (Reyes-Becerril et al., 2016). A large number of diverse basidiomycetous yeasts have been isolated from soil of different geographic regions, including the tropical and arctic zones (Tekolo et al., 2010). Some of the basidiomycetous yeasts enter the soil from plant materials, while others are observed in marine waters (Atlas and Bartha, 1981). Recently, some strains of salt-tolerant basidiomycetous yeasts, such as *S. halophilus* were also successfully isolated from the gut symbionts of a wood-feeding termite (WFT) species, *Reticulitermes chinenesis* in our laboratory (Ali et al., 2017). However, reports on the biodiversity and eco-physiology of salt-tolerant yeasts, adaptation mechanisms under hypersaline conditions, as well as their biotechnological potentials are still limited (Haliru et al., 2018).

Exposure of non-adapted microorganisms to hypersaline conditions, results in adverse effects such as cell shrinkage. Hence, some adaptive mechanisms must be acquired by these organisms to counteract the denaturing effect of high NaCl concentration, as well as to survive in extremely saline conditions (Zajc et al., 2012). Numerous morphological adaptations reflected by salt-tolerant yeasts have been reported, including change in the composition of the cell wall and cytoplasmic membrane, pigmentation, and meristematic growth. The main physiological responses of salt-tolerant yeasts to a high NaCl concentration included accumulation of compatible solutes, production of polysaccharide and the synthesis of intracellular sodium/potassium content (Haliru et al., 2018). Since salt-tolerant basidiomycetous yeasts, such as *Sterigmatomyces* can successfully survive in an environment of low *a*_w_ together with a combination of other extreme conditions, their robustness in the harsh environmental condition is utmost in the search for novel biotechnological applications of salt-tolerant yeasts in wastewater treatment, food industry, biofuel production, agriculture, environmental bioremediation, and oleochemicals industry.

Enzymes from salt-tolerant yeasts have been reported to be active in the occurrence of organic solvents due to their activity under a low *a*_w_ reaction system. Thus, the salt-tolerant yeast lipase-producing *Candida antarctica*, has a great application in biodiesel production (Sana, 2015). Bioethanol production has further increased the need to improve multistress tolerance of yeasts involved in industrial production of bioethanol as a result of the osmotic stress factor amongst other factors that significantly decrease bioethanol yields (Gostincar et al., 2011). Halotolerant yeasts have been reported to produce various metabolites of industrial interests such as antibiotics, terpenes, amino acid derivatives, and compatible solutes (e.g., glycerol, hydroxyacetone, ectoine, and trehalose) (Uratani et al., 2014). On the other hand, salt-tolerant yeasts degrade both aliphatic and aromatic hydrocarbons by using them as a carbon and energy source for metabolism (Uratani et al., 2014). Compared to bacteria, salt-tolerant yeasts are the preferred choice for the industrial generation of nanoparticles because they generally release large quantities of unique enzymes and biomolecules and are capable of growing fast in a relatively simple and inexpensive media (Mohite et al., 2015).

Exploration of newly isolated yeasts from WFT symbionts toward the degradation of azo dyes under high salt conditions is gaining attention, making the treatment of textile dyes by halotolerant yeasts a novel biodegradation approach in these habitats. Therefore, this study aims to evaluate the performance of decolorization and biodegradation on various azo dyes made by a newly isolated yeast *S. halophilus* SSA-1575 identified from *R. chinenesis*, where our attention was particularly focused on its degradation efficiency, involved mechanism, as well as its detoxification capability under a high-salt condition. The findings in this study are expected to provide an efficient biological strategy valued for the treatment of dyeing effluents containing azo dyes and a high load of salts.

## Materials and Methods

### Azo Dyes and Culture Medium

Five azo dyes namely, Reactive Black 5 (RB5), Reactive Red 120 (RR120), Reactive Blue 19 (RB19), Acid Scarlet GR (AS-GR), and Azure B (AzB) were used in this study (Table 1). A Minimal Salt (MS) medium was used for isolating halotolerant azo-degrading yeast strains. This medium contained (per liter of distilled water) 20 g NaCl, 10 g glucose, 1.0 g KH_2_PO_4_, 0.2 g (NH_4_)_2_SO_4_, 0.05 g CaCl_2_, and 0.05 g MgSO_4_. The pH of the MS medium was adjusted to 5 and autoclaved at 115°C for 30 min after adding the corresponding azo dyes.

**Table 1:** Performance of *S. halophilus* SSA-1575 on textile dye degradation.

**Azo dye**	**Molecular structure**	**Agar plate**		**Liquid medium**			**Maximum decolorization (%)**
		**Before decolorization**	**After decolorization**	**Before decolorization**	**After decolorization**		
					**Suspension**	**Supernatant/cell pellets**	
RB5	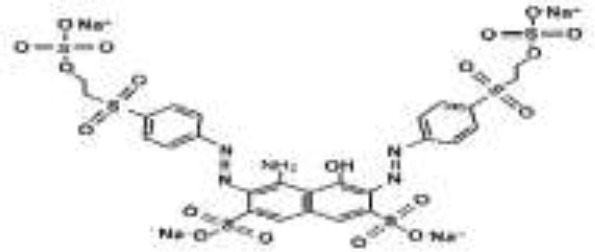	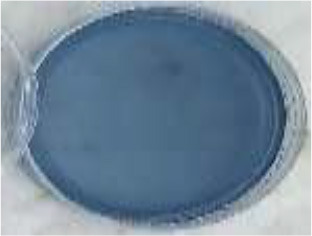	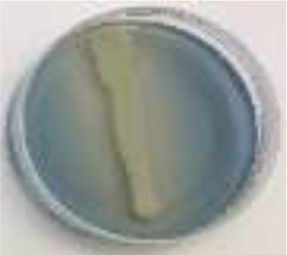	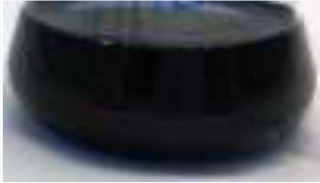	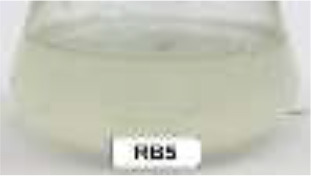	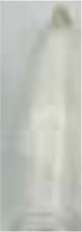	98.71
RR120	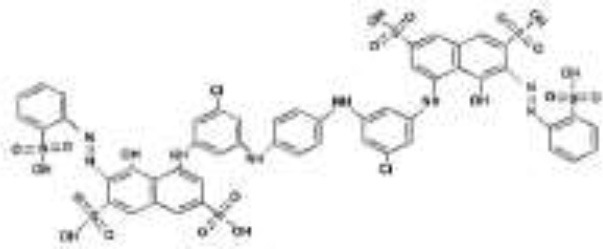	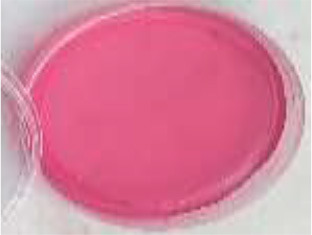	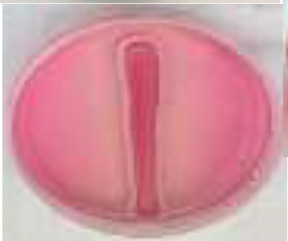	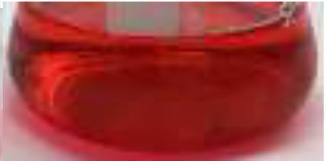	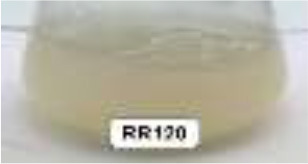	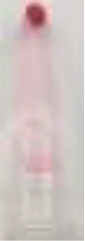	93.46
RB19	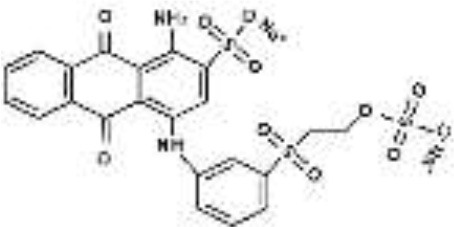	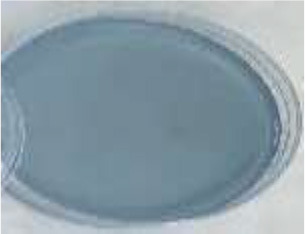	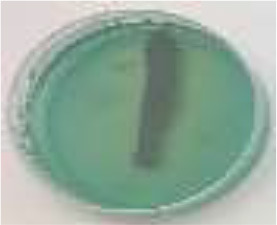	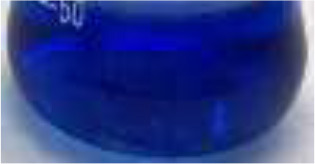	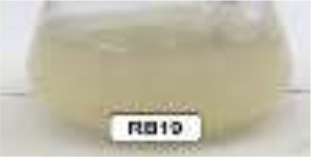	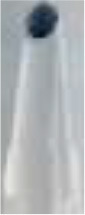	94.80
AzB	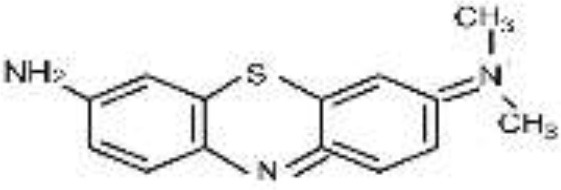	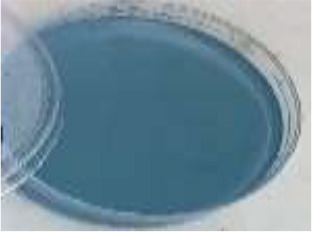	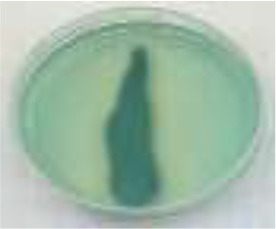	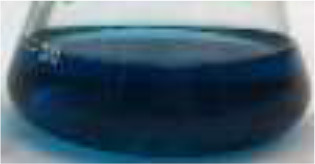	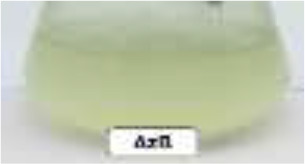	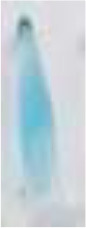	95.88
AS-GR	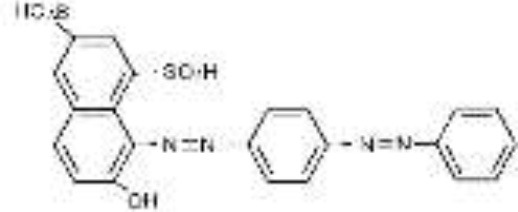	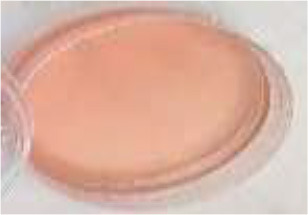	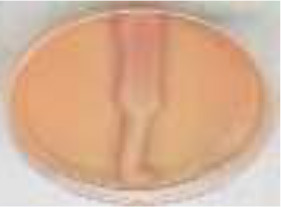	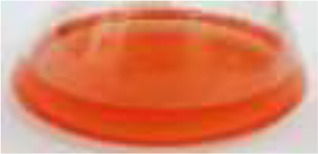	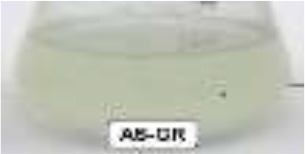	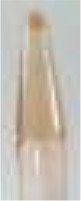	96.56

*RB5, Reactive Black 5; RR120, Reactive Red 120; RB19, Reactive Blue 19; AzB, Azure B; AS-GR, Acid Scarlet GR*.

### Screening, Enrichment and Characterization of Dye-Decolorizing Yeast

**Figure 1:**
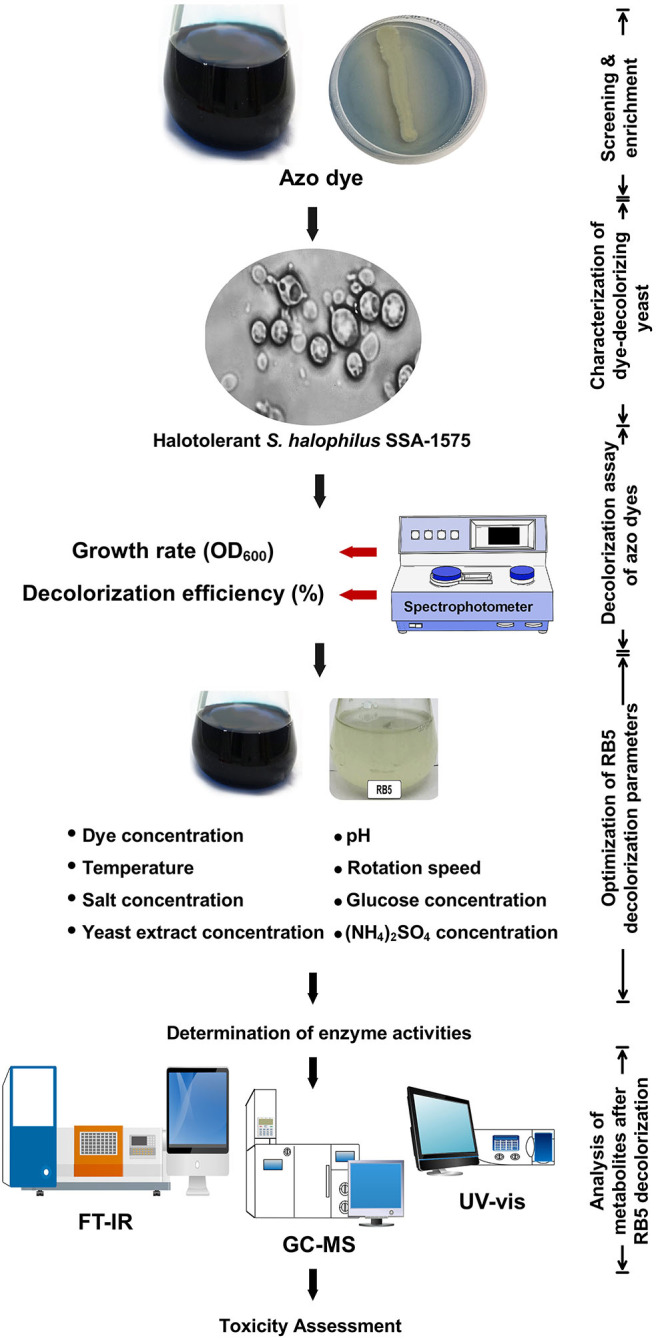
Experimental design used in screening halotolerant yeasts capable of decolorizing and detoxifying azo dyes.

In order to screen yeasts capable of decolorizing and detoxifying azo dyes, the experimental set-up used during this study is depicted in Figure 1. As per our previous study (Ali et al., 2017), *R. chinenesis* was used as a biological source for the isolation of azo dye-decolorizing yeasts. Aliquots of crushed WFT gut solutions (10 mL) were inoculated into a 250 mL Erlenmeyer flask containing 100 mL MS broth medium amended with different azo dyes (Table 1) at an initial concentration of 50 mg/L and incubated at 30°C under static condition. Once decolorization was observed, a 10 mL culture was further inoculated into a fresh dye-containing MS broth medium for another round of enrichment. The procedures were subsequently repeated 10 times until the decolorization efficiency was kept stable. The capability of the enriched yeast strains to decolorize different azo dyes was also examined similarly on agar plates containing 50 mg/L dye and the streaked plates were incubated at 30°C for 48 h. Abiotic controls (without yeast inoculation) were always included. The fastest-growing colonies capable of decolorizing various azo dyes and showing a high ratio of a zone of decolorization to a colony diameter, were isolated for morphological and physiological characterization as previously reported (Ali et al., 2017). As a result, a yeast strain designated as SSA-1575 was selected for further experiments. Genomic DNA extraction, purification, and primers used were also described in detail (Ali et al., 2017, 2019). Sequences were aligned and compared with sequences available at BLAST-n site in the GeneBank database. A phylogenetic tree was constructed by MEGA 7.0 using the neighbor-joining method (Saitou and Nei, 1987).

### Decolorization Assay of Azo Dyes

Dye decolorization assays were performed following Ali et al. (2019), with minor modifications. Briefly, 5% (v/v) of yeast culture (OD_600_ of 0.2) inoculation size was inoculated in 100 mL Erlenmeyer flasks containing 50 mL MS medium plus 50 mg/L of the tested azo dye and incubated at 30°C for 24 h. An aliquot (2 mL) of each culture medium was withdrawn at different time intervals. The aliquot was centrifuged at 12,000 rpm for 3 min to separate cell mass. The supernatant was used to determine the decolorization by measuring its absorbance with a UV-Vis spectrophotometer at the following maximum wavelength (λmax): RB5 (λ_max_ 595 nm), RR120 (λ_max_ 537 nm), RB19 (λ_max_ 592 nm), AzB (λ_max_ 650 nm) and AS-GR (λ_max_ 511 nm). The cell growth was also measured in terms of the difference between the optical density (OD) of the collected cell suspension before and after centrifugation at λ_max_ 600 nm (Guo et al., 2020).

### Optimization of RB5 Decolorization Parameters

The effect of different physico-chemical parameters on the decolorization of RB5 by growing cells of *S. halophilus* SSA-1575 was analyzed. The dye concentration was adjusted by changing the initial concentration of RB5 in the MS medium to 0.0, 50, 100, 250, 500, 750, 1,000, 1,500, and 2,000 mg/L. The salt concentration was adjusted by adding different concentrations of NaCl (0.0, 10, 20, 30, 40, 50, 60, 70, 80, 90, and 100 g/L) to the medium. The decolorization efficiency was also measured under different temperature (10–60°C), rotation speed (0.0, 50, 100, 150, and 200 rpm) and pH values (3, 4, 5, 6, 7, 8, 9, and 10). The concentrations of carbon and nitrogen sources were adjusted by adding glucose (0.0, 1, 2, 3, 4, 5, 6, and 7 g/L), (NH_4_)_2_SO_4_ (0.0–1.0 g/L) and yeast extract (0.0–1.0 g/L) to the medium.

### Determination of Enzyme Activities

The activities of Lac, LiP, and MnP, azoreductase, NADH-DCIP reductase, xylanase, and endoglucanase (CMCase) were monitored spectrophotometrically in cell free extracts as well as in the control supernatant using a UV-Vis spectrophotometer. After decolorization process, a 15 mL cell suspension was collected and centrifuged at 5,000 rpm for 15 min. The cell free extract was used for the determination of extracellular enzymes. However, for determining the activities of intracellular enzymes, the supernatant of sonicated cells was used. The activities of Lac, MnP, and LiP were determined using 2,2-azonodi-3-ethylbenzothiazoline-6-sulfuric acid (ABTS), 2,6-dimethoxyphenol (2,6-DMP), and veratryl alcohol, respectively, as the corresponding substrates following the previous methods (Tekolo et al., 2010; Ali et al., 2020a). The azoreductase activity was assayed by determining the decrease in the methyl red concentration at 440 nm (Kalyani et al., 2009). NADH–DCIP reductase activity was assayed at 590 nm following the method described by Saratale et al. (2009). The estimation of total reducing sugars (Miller, 1959) was also performed for determining the activities of xylanase and endoglucanase (CMCase) based on the 3,5-dinitrosalicylic acid (DNS) reagent (Bailey et al., 1992) using beechwood xylan and CMC as the corresponding substrates as previously described (Ali et al., 2020b).

### Analysis of Metabolic Intermediates After Dye Decolorization

RB5 dye before and after its decolorization by *S. halophilus* SSA-1575 was monitored using a UV-Vis spectrophotometer over a UV-Vis region spectrum (200–700 nm). The supernatants were also analyzed by the Fourier Transformed Infrared (FTIR) spectrum using a Bruker-Tensor 27 FTIR spectrophotometer (Bruker-Tensor 27), between a transmittance range of 400 and 4,000 cm^−1^ (Kenawy et al., 2019). To predict possible mechanisms of the decolorization process, the cell free extracts were analyzed by Mass Spectrometry for detecting metabolites formed after dye biodegradation (Ali et al., 2019). The injector temperature was set at 250°C.

### Toxicity of RB5 and its Metabolites After Decolorization

It is of great concern for any bioremediation technology to evaluate the toxicity of original dyes and their metabolic products after decolorization. The toxicity of RB5 and products obtained after its degradation by *S. halophilus* SSA-1575, has been assessed with respect to acute toxicity that have been assessed *via* the Microtox test using *Vibrio fischeri* (García-Montaño et al., 2008).

### Microtox Assay

The acute toxicity of biotransformation metabolites was performed using the Microtox bioassay following the standard protocol (ISO 11348-3, 2007). Microtox test depends on measuring the reduction in the light amount emitted by *V. fischeri* after exposing this bioluminescent marine bacterium to RB5 for 30 min because of a disruption in the respiration process. Acute toxicity was expressed in terms of the Inhibition Ratio (IR) of the bacterial luminescence during the time of exposure to RB5. IR was calculated using the equation below:

$$\begin{array}{r} \text{IR}\left(\text{\%}\right)={\text{L}}_{0}\times {\text{R}}_{f}-{\text{L}}_{t}/{\text{L}}_{0}\times {\text{R}}_{f} \end{array}$$

L_0_; is the luminescence intensity at 0 min,

L_*t*_; is the luminescence intensity at t min,

R_*f*_; is the luminescence intensity of negative control (0-t min).

### Statistical Analysis

Results were analyzed statistically using Minitab 17.1.0.0 software (Minitab Inc., Pennsylvania, USA) and SigmaPlot Software 12.5.0.38 (SigmaPlot, Systat Software Inc., UK). The normality of data was estimated by the Shapiro-Wilk test. Comparisons between two or more groups were performed using ANOVA with Tukey-Kramer comparison test. Simple linear regression analysis was performed to evaluate the effect of dye and its metabolic products after decolorization on the viability of cells using a regression equation for prediction. The *p* < 0.05 is considered significant.

## Results and Discussion

### Identification and Characteristics of *S. halophilus* SSA-1575

Among xylanase-producing yeasts isolated from the gut symbionts of *R. chinenesis* (Ali et al., 2017), *S. halophilus* SSA-1575 could efficiently decolorize various azo dyes (Table 1). The majority of *S. halophilus* strains were commonly isolated either from adjacent environments or from marine habitats (Fell, 2011). However, strain SSA-1575 was first identified in an insect gut system. WFTs and their gut symbionts have co-evolved into an efficient mini-bioreactor, including a suite of specialized enzymes that synergistically have a unique capability on the decomposition of lignin-derived compounds, making them the most abundant and sustainable solar batteries on earth (Sun et al., 2014).

**Figure 2:**
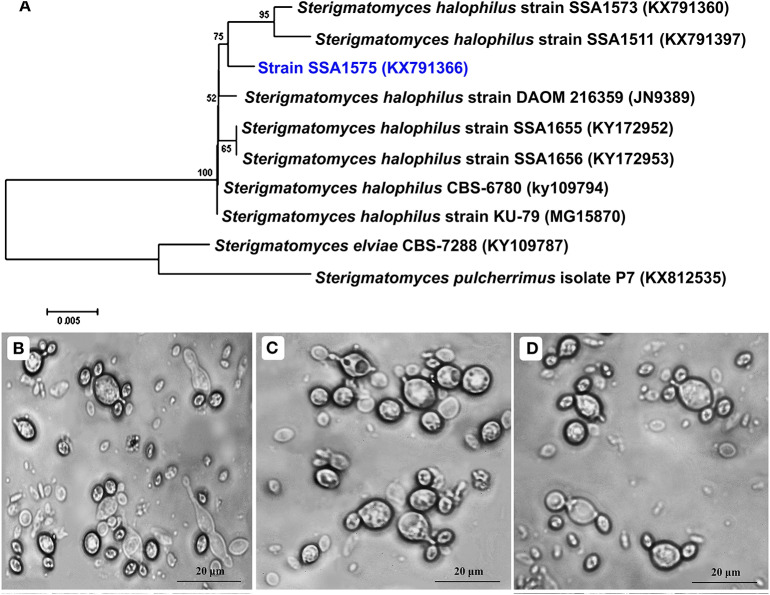
Identification and characterization of a newly isolated yeast strain, *Sterigmatomyces halophilus* strain SSA-1575, could efficiently decolorize various textile azo dyes. Neighbor-Joining phylogenetic tree of *S. halophilus* SSA-1575 with its closely related taxa. Bootstrap values from 1,000 replicates are shown at each branch for values >50%. GeneBank accession numbers are mentioned in parentheses. Scale bar represents 0.005 Jukes-cantor distances **(A)**. Cell morphology of strain SSA-1575 showing blastoconidia on stalk-like conidiophores. Yeast cells after 3 days **(B)** and 7 days **(C)** in YMB and YMA, respectively at 30°C. True hyphae and pseudohyphae are absent after 10 days of growth on CMA **(D)**.

To taxonomically identify strain SSA-1575, the D1/D2 domain and ITS regions were amplified and the sequence was deposited in the GeneBank with the accession number KX791366. BLAST analysis proposed that the *S. halophilus* strain SSA-1575 was relevant to the *S. halophilus* strain SSA1511 (accession number KX791397) with an identity of 98% in the D1/D2 region (Figure 2A). To morphologically identify strain SSA-1575, the growth in Yeast Malt Broth (YMB) and on Yeast Malt Agar (YMA), as well as Dalmau plate growth on Corn Meal Agar (CMA) was examined microscopically (Figures 2B–D). The yeast colonies were chalky-white with a rough surface and an entire margin on YMA plates after 7 days at 30°C. The cells were mostly spherical to ovoid with blastoconidia that occurred singly or in clusters on short conidiogenous stalks. True hyphae and pseudohyphae are absent after 10 days of growth on the CMA at 30°C. The physiological and biochemical characters used for the identification of strain SSA-1575 were also performed (Table 2).

**Table 2:** Key morphological and physiological characteristics of *S. halophilus* strain SSA-1575.

**Traits**		**Traits**		**Traits**		**Traits**	
**Fermentation**							
D-galactose	–	Cellobiose	+/–	Soluble starch	–	Glucose	+
Maltose	–	Sucrose	–	Lactose	+	Melezitose	–
α, α-trehalose	+/–	Inulin	–	D-xylose	+	Raffinose	–
Melibiose	–						
**Carbon assimilation**							
D-glucose	+	D-galactose	–	D-xylose	+	Maltose	–
Sucrose	–	Cellobiose	–	Trehalose	+	Lactose	+
L-rhamnose	–	Salicin	–	Melibiose	–	Inulin	–
D-arabinose	+/–	Raffinose	–	Melezitose	–	Galactitol	–
L-arabinose	+	Ribitol	+	D-glucitol	–	D-gluconate	–
D-mannitol	+	*myo*-inositol	–	N-acetyl-D-glucosamine	–	Methanol	+/–
2-keto-D-gluconate	–	Succinate	+	Glycerol	+	DL-lactate	–
Ethanol	+	Erythritol	–	Citrate	+	Soluble starch	–
L-sorbose	–	D-ribose	+	Arbutin	–	D-glucosamine	–
**Nitrogen assimilation**							
Nitrate (potassium)	+	L-lysine	–	Creatinine	–	Ethylamine	–
Nitrite (sodium)	–	Cadaverine	–	Imidazole	–	Creatine	–
**Vitamin requirements**							
Vitamin-free	+/–						
**Growth tests**							
25°C	+	30°C	+	35°C	+	37°C	+
Acetic acid (1%)	+	NaCl (10%)	+	NaCl (16%)	+	Diazonium blue B	+
D-glucose (50%)	+	D-glucose (60%)	+	Acetic acid production	+	Urea hydrolysis	+/–

*+; positive, –; negative, +/–; variable*.

### Performance of *S. halophilus* SSA-1575 on Azo Dye Decolorization

The growing cells of *S. halophilus* SSA-1575, were recorded for relatively high potential in dye decolorization performance under static conditions (Table 1), while it could not decolorize these dyes well in an anaerobic environment (i.e., under an O_2_-free nitrogen atmosphere) (data not shown). On the other hand, a sharp decline in decolorization efficiency was observed under agitation conditions. It is concluded that *S. halophilus* SSA-1575, may require a certain amount of oxygen in accordance with that reported by Bor-Yann (2002). The decolorization efficiency of tested azo dyes by SSA-1575, at the dye concentration of 50 mg/L was more than 93% within 12–21 h. RB5 was decolorized mainly through the biodegradation process. However, both suspension and cell pellets are still colored, even the decolorization efficiencies for RR120, AzB, and AS-GR are apparently high, suggesting that these dyes might be decolorized through adsorption only or the combined effects of biodegradation and adsorption. Though the color of RB19 was probably removed through the effect of the adsorption process only, it has just accumulated but not decomposed. It has been reported that *Candida tropicalis* strain Y2-0814, was effectively decolorized 100 mg/L RB5 within 24 h through biodegradation (Yang et al., 2003), while *C. tropicalis* could effectively decolorize RB5 through the adsorption process (Dönmez, 2002).

As far as it is known, very few studies are conducted for halo-tolerant fungi capable of dye decolorization. To date, no report has been published on the decolorization of azo dyes by yeast species belonging to *S. halophilus*. The highest decolorization efficiency performed by *S. halophilus* SSA-1575, in a relatively short time suggested its valuable potential in the treatment of industrial effluents containing a wide variety of azo dyes at a high salt environment.

### Optimization of Dye Decolorization Efficiency

**Figure 3:**
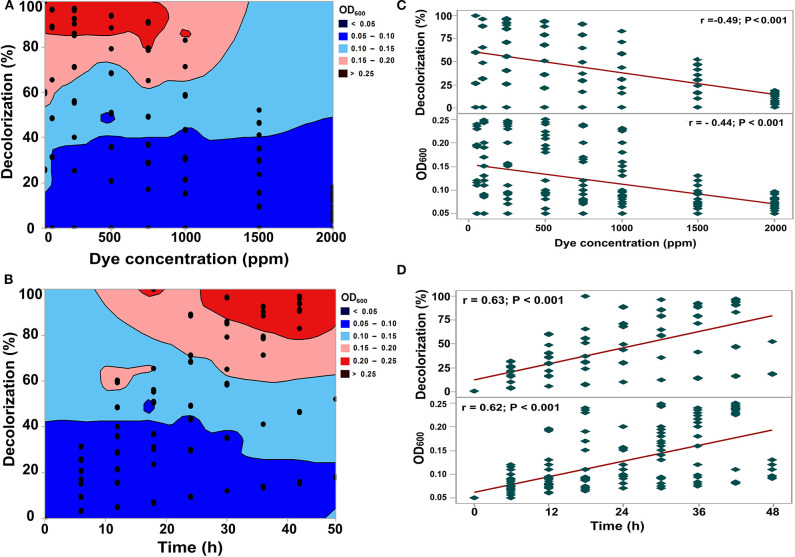
Effect of dye concentration and time on the decolorization efficiency of RB5 and growth rate of *S. halophilus* SSA-1575 cells as represented by contour plot graphs **(A,B)** and correlations **(C,D)**. *p* < 0.05 is considered significant.

**Figure 4:**
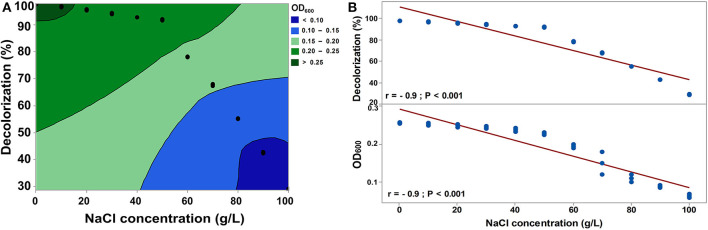
Effect of salt concentration on the decolorization efficiency of RB5 and growth rate of *S. halophilus* SSA-1575 cells, as represented by contour plot graphs **(A)** and correlations **(B)**. Dye concentration was fixed at 50 mg/L. *p* < 0.05 is considered significant.

**Figure 5:**
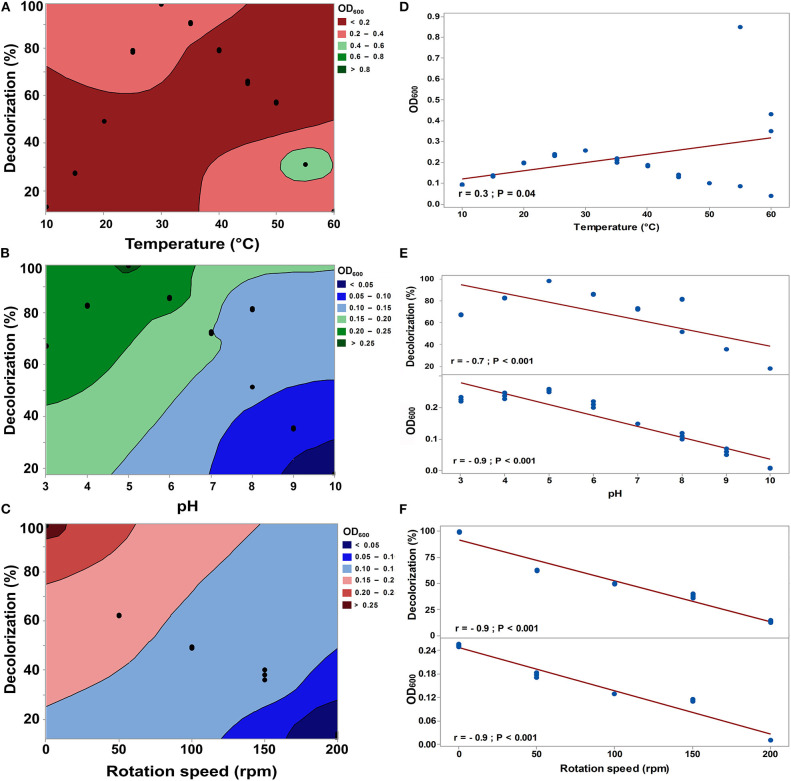
Effect of temperature, pH, and rotation speed on the decolorization efficiency of RB5 and growth rate of *S. halophilus* SSA-1575 cells, as represented by contour plot graphs **(A–C)** and correlations **(D–F)**. Dye concentration was fixed at 50 mg/L. *p* < 0.05 is considered significant.

**Figure 6:**
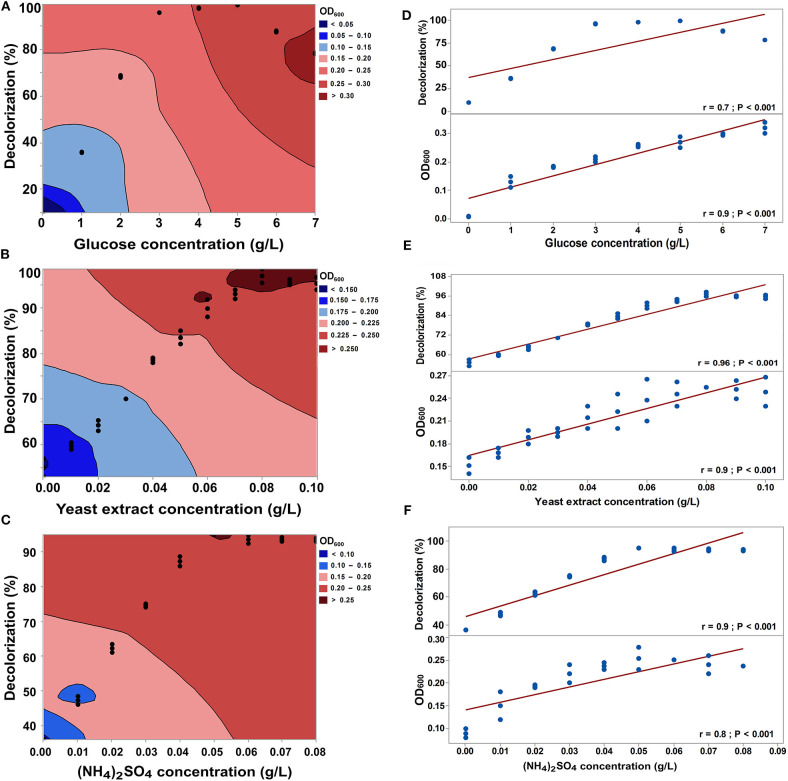
Effect of glucose concentration, yeast extract concentration and (NH_4_)_2_SO_4_ concentrationpH and rotation speed on the decolorization efficiency of RB5 and growth rate of *S. halophilus* SSA-1575 cells, as represented by contour plot graphs **(A–C)** and correlations **(D–F)**. Dye concentration was fixed at 50 mg/L. *p* < 0.05 is considered significant.

Various physicochemical parameters, including dye concentration, salt concentration, pH, temperature, rotation speed, glucose, ammonium sulfate, and yeast extract, were evaluated in terms of the decolorization performance on RB5 dye by *S. halophilus* SSA-1575 (Figures 3–**6**). Different techniques, such as direct counts using a microscope, colony counts, biomass measurement, or light scattering (turbidity of a culture in a spectrophotometer) have been used for monitoring microbial growth rate. However, the determination of turbidimetry may be considered the most widespread analytical tool to determine microbial growth, in which turbidimetry is a fast and non-destructive method (Maia et al., 2016).

The effects of the initial RB5 concentration and time on the growth of *S. halophilus* SSA-1575 and its decolorization efficiency were shown in Figures 3A,C, respectively. Clearly, the decolorization efficiency reached 100% at 50 mg/L RB5 within 24 h by growing cells of SSA-1575. Increasing of the RB5 initial concentration has resulted in a significant increase in its reaction time for approaching maximal decolorization percentage, which was marked with a decrease in its growth rate (*p* < 0.001). Significant negative correlations between the dye decolorization and the growth rate with dye concentration were observed, while the direction of the linear relationship was significantly positive with the decolorization time (Figures 3B,D). The decreased decolorization efficiency with incubation time was probably due to an increase in toxicity of RB5, with its biotransformation metabolites on growing yeast cells, or even possible depletion of nutrients and redox mediators (Chang et al., 2001; Isik and Sponza, 2003; Dafale et al., 2008; Agrawal et al., 2014). Meanwhile, maximum decolorization efficiency (>90%) was observed at 100–750 mg/L within 18–42 h. Then, a significant reduction in the decolorization efficiency (*p* < 0.001), which reached 83, 52 and 18% at 1,000, 1,500, and 2,000 mg/L RB5, respectively. Simultaneously, the growth rate of SSA-1575 cells was decreased within 42–48 h by increasing the dye concentration from 1,000 to 2,000 mg/L. Compared with other salt-tolerant yeasts isolated from sea mud; *Pichia occidentalis* strain G1 and *Scheffersomyces spartinae* strain TLHS-SF1 (Tan et al., 2016; Song et al., 2017), the newly isolated yeast *S. halophilus* SSA-1575 showed a competitive decolorization performance.

The effect of salt concentration on RB5 decolorization by strain SSA-1575, as well as its growth rate is depicted in Figure 4A. SSA-1575 showed a decolorization efficiency of more than 92% under the effect of a 50 mg/L RB5 and NaCl concentration of up to 50 g/L. However, a further increase in the salt concentration above 50 g/L showed an observable decrease in the decolorization efficiency by *S. halophilus* SSA-1575, to <79%. Another reason for the reduction of dye removal at high salt concentrations, could be the decrease in dye solubility. On the other hand, the growth rate of strain SSA-1575 at 0–50 g/L NaCl concentration, was significantly better than any higher salinity when beyond 50 g/L, suggesting that *S. halophilus* SSA-1575 is a salt-tolerant yeast strain, instead of a halophilic one (Woolard and Irvine, 1995). Figure 4B revealed significant negative correlations between the dye decolorization and the growth rate with a salt concentration. The results of decolorization efficiency revealed by SSA-1575 were obviously much better than those obtained by Tan et al. (2016). Hence, *S. halophilus* SSA-1575 could be a more competitive azo-degrading yeast if compared with other salt-tolerant yeasts reported in literature.

Temperature variation had a significant influence on the decolorization efficiency of RB5 by *S. halophilus* SSA-1575 and its growth (Figure 5A). Over 98% of dye decolorization was achieved at 30°C under the static condition. A further increase in temperature to above 30°C led to a decrease in decolorization efficiency, reaching only 11.2% decolorization efficiency at 60°C, which was probably due to the loss of cell viability (Jadhav et al., 2008). The broad range of temperature makes *S. halophilus* SSA-1575 more suitable for dye bioremediation. As depicted in Figure 5B, the decolorization efficiency of RB5 (50 mg/L) by *S. halophilus* SSA-1575 was achieved at a broad range of pH values (3.0–10.0), with the optimum being pH 5, since over 98% dye decolorization efficiency was achieved at this pH value. Meanwhile, a significant decrease in decolorization percentages of RB5 at strongly acidic or alkaline pH conditions (*p* = 0.003) was observed. The change of pH value may affect the solubility of RB5 in water, as well as the reductive activity of *S. halophilus* SSA-1575 cells during the dye decolorization process. The fact that *S. halophilus* SSA-1575 could decolorize RB5 in a relatively wide range of pH values, makes it a potent yeast candidate for dye removal from textile wastewater effluents or dyeing mills that differ significantly in pH (Saratale et al., 2013). Generally, dye concentrations in textile effluents vary from 10 to 25 mg/L (El Bouraie and El Din, 2016). RB5 is one of the most commonly used reactive dyes in the dyeing industry, since it is a highly soluble synthetic dye in water (Asad et al., 2007; Vijaykumar et al., 2007).

Agitation was another important parameter that affected the dye decolorization efficiency by *S. halophilus* SSA-1575 and its growth rate (Figure 5C). Over 98% decolorization efficiency of RB5 by SSA-1575 strain was achieved under the static condition. However, a significant (*p* = 0.009) sharp decrease in the decolorization efficiency was obtained under the agitation condition (50–200 rpm), reaching 13% at an agitated condition of 200 rpm. The negative results for the rotation speed factor on RB5 decolorization, could probably be due to the competition between oxygen and azoreductase enzyme (Kumar et al., 2019). These results are actually in agreement with some references reported by Agrawal et al. (2014) and Wang et al. (2009), who found that only 9 and 13% Acid Black 210 and Reactive Red 180 were decolorized by *Providencia* sp. strain SRS82 and *Citrobacter* sp. strain CK3, respectively, when the rotation speed was established at 150 rpm. A significant decrease in the growth rate was further recorded along with a rotation speed increase from 50 to 200 rpm (*p* = 0.008). It has been reported that a reduced agitation speed (<200 rpm) significantly repressed the growth of *Bacillus* sp. strain DRS-1, as a result of a deficiency in aeration and nutrient availability (Kalme et al., 2007; Jadhav et al., 2008; Vijayanand et al., 2010). Agitation speeds that were higher or lower than 100 rpm led to a reduction in the growth of *Bacillus licheniformis* BT5.9 and its enzymatic production (Ibrahim et al., 2013). At high agitation speeds, the greater shear forces led to a higher rate of cell destruction (Venkatadri and Irvine, 1990). The correlations of decolorization efficiency of RB5 by strain SSA-1575 and its growth rate at various parameters, including temperature pH and rotation speed were presented (Figures 5D–F). A significant positive correlation between the temperature and growth rate of strain SSA-1575 was recorded, while a significant negative correlation was shown between the dye decolorization and the growth rate with pH and rotation speed.

Figure 6A shows the effect of glucose concentration on the RB5 decolorization by SSA-1575, as well as its growth rate. More than 95% of 50 mg/L RB5 was decolorized by *S. halophilus* SSA-1575 when the glucose concentrations were 3.0–4.0 g/L. Meanwhile, the decolorization efficiencies were significantly decreased (*p* = 0.009) all <90% when the concentrations of glucose were found to be 0.0–2.0 g/L and 6.0–7.0 g/L. Hence, neither inadequate nor excessive amounts of glucose would be of benefit for RB5 decolorization by the growing cells of *S. halophilus* SSA-1575. These findings are in accordance with that obtained by Song et al. (2017). *S. halophilus* SSA-1575As could not utilize RB5 as the sole source of carbon and energy. Typically, azo dyes have low carbon content, which makes biodegradation extremely difficult (Khelifi et al., 2009; Eskandari et al., 2019). Therefore, adding adequate external carbon sources is always necessary for effective dye decolorization (Jamee and Siddique, 2019). Glucose was found to be an ideal candidate in optimizing dye decolorization efficiency (Khelifi et al., 2009; Song et al., 2017; Eskandari et al., 2019). However, when the proportion of external carbon source to dyes exceeds a certain range, microorganisms would prefer to utilize external carbon rather than dyes (Qu et al., 2012). Hence, decolorization efficiency can significantly drop down at an excessive amount of glucose higher than 5 g/L. In contrast, a higher concentration of glucose was found to increase the growth rate of *S. halophilus* SSA-1575, which concluded that the effect of glucose concentration on RB5 decolorization by strain SSA-1575 and its growth rate were different as previously reported (Tan et al., 2016; Song et al., 2017).

The efficiency of RB5 decolorization (50 mg/L) by strain SSA-1575 increased significantly from 55 to 97% (*p* < 0.001) in the presence of low concentrations of yeast extract (<0.08 g/L) (Figure 6B). At this optimal concentration, the decolorization efficiency of RB5 by *S. halophilus* SSA-1575 was 97% and this could corroborate the role of yeast extract, as a nitrogen source, in enhancing the growth and metabolic activities of some yeasts (Sitepu et al., 2014). No significant increase in the decolorization efficiency or growth rate was observed above 0.08 g/L, which confirmed that at this concentration of yeast extract, both decolorization efficiency and growth rate were optimal. On the other hand, the decolorization efficiency was significantly increased from 36 to 95% (*p* = 0.002) when the concentration of (NH_4_)_2_SO_4_ reached its optimal concentration of 0.05 g/L (Figure 6C). The direction of the linear relationship was significantly positive with glucose concentration, yeast extract concentration, and ammonium sulfate concentration (Figures 6D–F). Clearly, a significant positive correlation (*r* = 0.63; *p* < 0.001) was also observed between the growth rate of SSA-1575 and its decolorization efficiency.

### Enzyme Activity and Performance

*S. halophilus* SSA-1575 could efficiently decolorize RB5 due to the involvement of its unique enzymatic system. To get further insight into the decolorization mechanism of RB5 by *S. halophilus* SSA-1575, the enzyme activities of Lac, MnP, LiP, reductases (azoreductase and NADH-DCIP reductase), CMCase and xylanase were evaluated (Table 3). A significant induction (*p* = 0.009) in NADH-DCIP reductase by 110% was observed when the activity was compared with control (with 0 mg/L NaCl). Meanwhile, azoreductase activity was significantly increased by 76% compared with the control (*p* = 0.005). The performance of *Kocuria rosea* MTCC1532 on the decolorization of methyl orange was improved as the addition of azo dye could induce azoreductase and NADH-DCIP reductase activities (Parshetti et al., 2010). These results suggested a capability of the yeast strain, *S. halophilus*SSA-1575, to produce various reductases that may potentially synergize the degradation processing at the high dye and salt concentrations. Several studies reported the role of azoreductase and NADH-DCIP reductase produced by fungi, yeasts, and bacteria in the reductive cleavage of -N=N- group of azo dyes under microaerophilic conditions (Liu et al., 2013; Yang et al., 2013; Song et al., 2017). On the other hand, a significant increase in CMCase and xylanase activities was noticed when compared with controls (*p* = 0.02 and < 0.001, respectively).

**Table 3:** Activities of enzymes produced by *S. halophilus* strain SSA-1575 after decolorizing 50 mg/L RB5 under low and high salinities.

**Enzyme**	**Control (with 0 g/L NaCl)**	**Test (with 30 g/L NaCl)**
NADH-DCIP reductase^a^	20.7 ± 0.04	43.35 ± 0.8*
Azoreductase^b^	2.83 ± 0.8	4.98 ± 1.2*
Lignin peroxidase^c^	0.452 ± 0.008	0.175 ± 0.003*
Laccase^c^	0.367 ± 0.002	0.193 ± 0.06*
Manganese peroxidase^c^	0.171 ± 0.006	0.078 ± 0.07
CMCase^c^	4.32 ± 1.4	8.81 ± 1.1*
Xylanase^c^	3.70 ± 0.07	7.378 ± 0.12*

Values are mean of triplicates ± SD. Significantly of enzyme activities after dye decolouration differ from target dye (control) at *P < 0.05 using one-way (ANOVA) with Tukey-Kramer comparison test.

a *Activity measured by μg of DCIP reduced/min/mg protein*.

b *Activity measured by μg of methyl red reduced/min/mg protein*.

c *Activity measured by U/min/mg protein*.

As given in Table 3, no induction of Lac, LiP, and MnP was found. The activities of Lac and LiP enzymes in the cell extracts of strain SSA-1575 were significantly decreased when compared with their controls (*p* < 0.001). MnP activity was also decreased over the controls but with non-significant differences. These results suggested that the activities of three LMEs were all inhibited by higher salinity which was different from azoreductase, NADH-DCIP reductase, CMCase, and xylanase. The activities of Lac and LiP in the cell extract of halotolerant yeast strain *Galactomyces geotrichum* GG were significantly reduced, indicating the inhibition of Lac and LiP by higher salinity (Guo et al., 2019). Similarly, the inhibition of salinity to the LMEs activities azo dye degrading microorganisms under high salt t condition was also reported previously (Hussain et al., 2013; Liu et al., 2017; Song et al., 2017). Compared with the results of the previous reports mentioned above, Zhao et al. (2014) demonstrated that LiP, Lac, and NADH-DCIP reductase were key bacterial enzymes produced by *Bacillus* sp. strain UN2 for the decolorization of methyl red. The fungal Lac and MnP enzymes were involved in the decolorization of textile effluents (Anastasi et al., 2012). The above results showed that the synergistic interactions between reductases and LMEs may result in outstanding dye decolorization performance by *S. halophilus* SSA-1575.

### Analysis of Intermediates Produced in the Decolorization of RB5 and the Proposed Pathway

**Figure 7:**
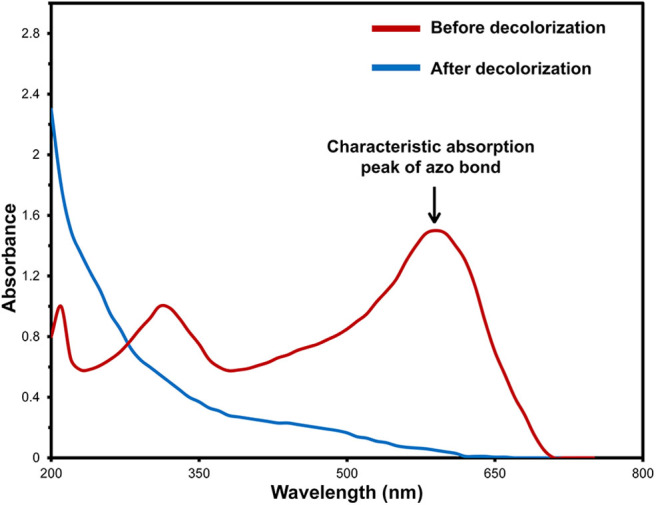
UV-vis spectral analysis of RB5 and its decolorization products from batch cultures of *S. halophilus* SSA-1575 containing 50 mg/L RB5 at different incubation times.

To understand well a possible degradation pathway of RB5 by the yeast strain SSA-1575, UV-Vis spectroscopy, FTIR and Mass Spectrometry analyses were employed. The absorbance spectra of RB5 before and after decolorization by *S. halophilus* SSA-1575 at different reaction times were presented (Figure 7). The maximum absorption peak of RB5 at 595 nm (visible region) was attributed to the presence of aromatic rings connected by the -N=N- bond. However, the absorption peak of RB5 at 310 and 203 nm (UV region) were attributed to benzene-like molecules. After dye decolorization, the intensity at 595 nm was sharply decreased to almost zero, which indicated the cleavage of -N=N- bonds (Enayatizamir et al., 2011; Álvarez et al., 2013; Agrawal et al., 2014; Tan et al., 2016; Martorell et al., 2017). Additionally, the intensities of the UV region disappeared. Differences in the maximum absorption of RB5 before and after decolorization by strain SSA-1575 provided evidence for the changes in the molecular structure of the dye, which was verified by the biotransformation metabolites detected after dye decolorization because its primary chromophores were attacked and then deconstructed (Dafale et al., 2008).

**Figure 8:**
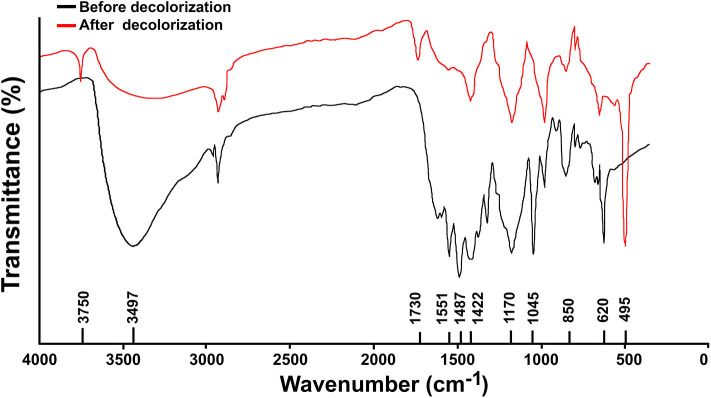
FT-IR analysis of RB5 and its decolorization products from batch cultures of *S. halophilus* SSA-1575 containing 50 mg/L RB5.

Significant changes were observed in the RB5 molecule after its decolorization by strain SSA-1575, which were indeed evidenced by the disappearance of some initial peaks and formation of some new peaks as confirmed by FTIR analysis (Figure 8). Peaks located at 1,540–1,400 cm^−1^ were assigned to -N=N- stretching of the asymmetric azo group, which was actually responsible for the dye color presence. The presence of peaks at 1,750–1,620, 3,400–3,250, 3,697–3,000, and 1,260–1,080 cm^−1^ confirmed the C-C stretching in the benzene ring, N-H stretching, O-H stretching and O-C stretching, respectively. The peak at 620 cm^−1^ also indicated N-H stretching. The Peak at 1,660–1,550 cm^−1^ showed NH_2_ bending. C-S and S-O stretching was observed at 1,260 cm^−1^. Peaks at 2,970–2,880, 1,250–1,140, 1,600–1,390, 1,070–1,010, and 1,130–1,070 cm^−1^ showed C-H asymmetric stretching in CH_2_ groups, R-SO_3_ group with =S=O stretching, N-H, alkenes, and sulfoxide SO stretching, respectively. Additionally, υ3 stretching and υ4 stretching of SO_4_ groups were located at peaks of 1,070 and 660–620 cm^−1^, respectively. The peak at 850 cm^−1^ indicated the vibrational mode of [HSO_4_]^−^ anion. Sulfone SO_2_ stretching was observed at 1,160–1,120 cm^−1^. Secondary amines were observed at 650–380 cm^−1^. The -N=N-, NH_2_, aromatic amines, C-C, sulfoxide SO and R-SO_3_ linkages showed a cleavage with prolonged decolorization reaction time, decrease in the dye color, confirming the cleavage of -N=N- bonds in RB5 (Yang et al., 2019). On the other hand, the new peaks generated after the dye decolorization, which were observed at 3,750 cm^−1^ (O-H stretching), 1,730 cm^−1^ (C=O stretching) and 1,500–1,400 cm^−1^ (C-C stretching) were clearly indicating the removal of amine from the degradation product (Yang et al., 2019). As a result of the appearance of new peaks in biotransformation metabolites and the disappearance of other peaks from the control spectrum, it is very clear that the yeast strain, *S. halophilus* SSA-1575, significantly deconstructed the molecular structure of azo dye RB5 during the decolorization process.

**Figure 9:**
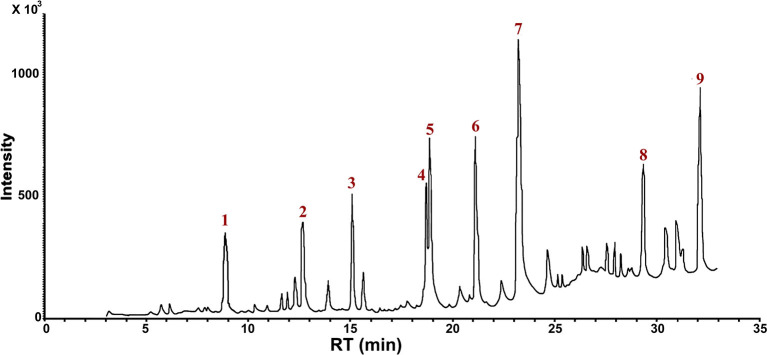
Mass Spectrometry analysis for Identification of metabolites after RB5 decolorization by *S. halophilus* SSA-1575.

**Figure 10:**
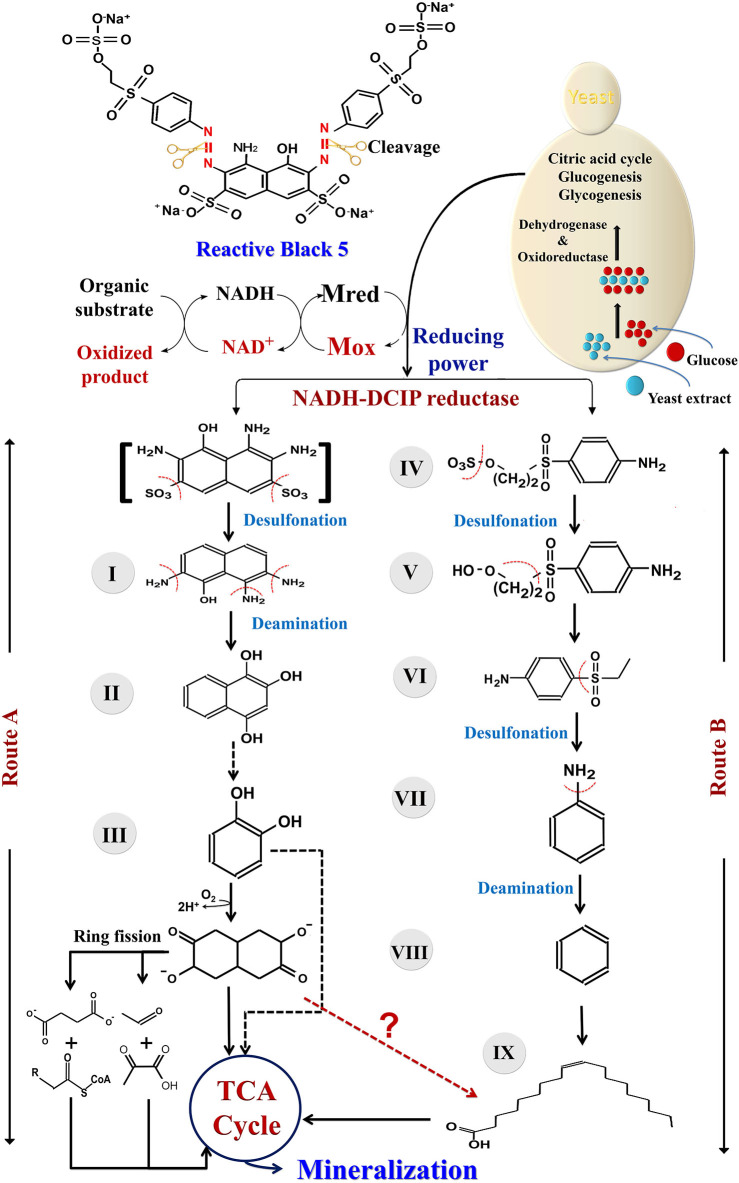
Proposed pathway for the degradation of RB5 by *S. halophilus* SSA-1575.

To further propose the possible degradation mechanism of RB5 dye by the newly isolated yeast strain *S. halophilus* SSA-1575, possible metabolic products were detected and identified using the Mass Spectrometry technique. As a result, nine possible metabolites were determined in terms of the corresponding mass spectra and *m*/*z* values of the extracted solution after the RB5 decolorization reaction (Table 4). Clearly, the mass spectra of the possible metabolites obtained after degradation of RB5 revealed major peaks at retention times (RT-min) 8.97, 12.66, 15.05, 18.70, 18.83, 21.08, 23.19, 29.30, and 32.90 min (Figure 9). Various mechanisms for azo dye biodegradation have been proposed in references. Generally, the degradation pathways follow two main routes: symmetrical or asymmetrical cleavage of azo bonds (Jain et al., 2012). Based on our investigation and an analysis of those byproducts and enzymes mentioned above, a possible pathway was proposed for the degradation of RB5 (Figure 10). The presence of glucose and yeast extract in the growth medium might have stimulated the expression of *S. halophilus* SSA-1575-producing oxidoreductases and dehydrogenases, which are active in glycolysis, gluconeogenesis and the TCA cycle. As a result, the generated reducing power may be transferred via the cytoplasmic membrane of the growing yeast cells, catalyzing reductase enzymes to reduce the azo bond of the RB5 dye. The reductive cleavage of the azo bonds was generally regarded as the first step toward azo dyes biodegradation (Song et al., 2018). It was obvious that *S. halophilus* SSA1575 has asymmetrical cleavage of RB5. Hence, the asymmetric cleavage of the RB5 azo bond was mainly catalyzed by NADH-DCIP reductase. The detection of one possible amine (2-((4-aminobenezene)sulfonyl)ethoxy)sulfonic acid (compound IV; RT 32.90 min; *m*/*z* 280) has further confirmed this speculation, however, the other amine 1,2,7-triamino-8-hydroxy-3,6-naphthalinedisulfonate (TAHNDS) was not monitored. The reason might be that TAHNDS is an unstable compound and it might be quickly degraded to other byproducts (Qu et al., 2012; Tan et al., 2016; Song et al., 2018), which was further confirmed by a detection of 2,7,8-Triaminonaphthalen-1-ol (compound I; RT 29.30 min; *m*/*z* 190). This compound was probably produced from the desulfonation of TAHNDS. Subsequently, both compounds I and IV, were probably further oxidatively transformed into smaller compounds through the routes A and B (Figure 10). As reported previously, aromatic amines produced during dye decolorization can be further degraded into smaller compounds and may eventually be mineralized (Oturkar et al., 2011; El Bouraie and El Din, 2016; Martorell et al., 2017; Zhuang et al., 2020). For route A, the compound I, which might be produced from the desulfonation of TAHNDS, was probably deaminated into naphthalene-1,2,4-triol (compound II; RT 23.19 min; *m*/*z* 176). Moreover, compound II was probably transformed into catechol (compound III; RT 8.97 min; *m*/*z* 109), which might be cleaved oxidatively into aliphatic metabolites *via* the cis-muconic acid pathway (Ferraroni et al., 2004; Oturkar et al., 2011). On the other hand, the ring fission of catechol was probably transformed into acetaldehyde, pyruvate, succinate, and acetyl-coA, which is the precursor of the TCA cycle (Schweigert et al., 2001). Moreover, compound III was probably transformed into 3,7-Dioxo-decahydronaphthalene-2,6-bis (olate), which might be mineralized directly *via* the TCA cycle or transformed into *cis*-9-Octadecenoic acid (compound IX; RT 12.66 min; *m*/*z* 281) before entering the TCA cycle. For route B, compound IV was probably further oxidatively transformed into three important intermediate compounds V, VI, and VII namely 2-((4-aminophenyl)sulfonyl)ethanol (RT 21.08 min; *m*/*z* 201), 4-ethanesulfonyl aniline (RT 18.70 min; *m*/*z* 184) and aniline (RT 15.05 min; *m*/*z* 93), respectively through desulfonation process. Compound VII was then transformed into another detected intermediate benzene (compound VIII; RT 18.83 min; *m*/*z* 78) *via* the deamination process. Notably, the degradation pathway of RB5 by *S. halophilus* SSA-1575 was proposed depending on the analysis of possible intermediate compounds and relevant literature (El Bouraie and El Din, 2016; Tan et al., 2016; Kale and Kane, 2018). Clearly, based on findings obtained by UV-Vis, FTIR, and Mass Spectrometry analyses, it could be concluded that the RB5 decolorization by strain SSA-1575 occurred *via* cleavage of the azo bond, resulting in the formation of colorless aromatic amines devoid of any chromophores (N=N).

**Table 4:** Compounds identified by Mass Spectrometry analysis after degradation of RB5 by *S. halophilus* strain SSA-1575.

**Peak No**.	**Compound name**	***m*/*z***	**RT (min)**
1	Catechol	109	8.97
2	*cis*-9-octadecenoic acid	281	12.66
3	Aniline	93	15.05
4	4-ethanesulfonyl aniline	184	18.70
5	Benzene	78	18.83
6	2-((4-aminophenyl)sulfonyl)ethanol	201	21.08
7	Naphthalene-1,2,4-triol	176	23.19
8	2,7,8-triaminonaphthalen-1-ol	190	29.30
9	(2-((4-aminobenezene)sulfonyl)ethoxy)sulfonic acid	280	32.90

### Toxicity Assessment for Environmental Safety

**Figure 11:**
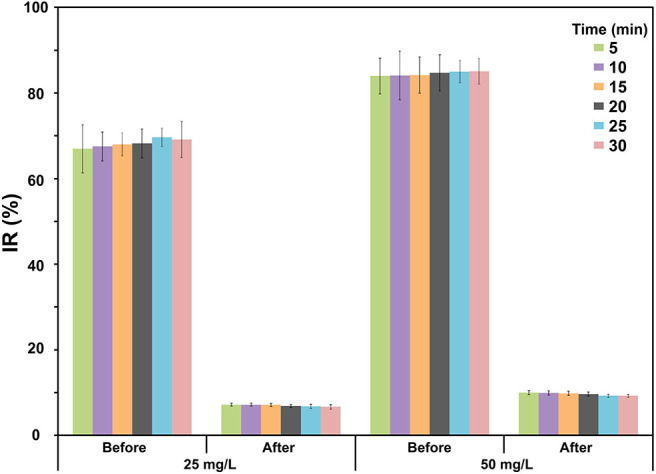
Acute toxicity of RB5 before and after its decolorization by *S. halophilus* SSA-1575 using Microtox bioassay, which indicated by Inhibition Ratio (IR) of luminescent *V. fischeri* after 30 min of exposure to RB5. High toxicity, 70 ≤ IR (%) < 100; moderate toxicity, 50 ≤ IR (%) <70; low toxicity: 20 ≤ IR (%) <50; micro toxicity, 10 ≤ IR (%) <20; non-toxicity, 0 ≤ IR (%) <10.

The ecotoxicology assessment of RB5 after a decolorization processing by *S. halophilus* SSA-1575, was finally conducted to evaluate the safety of its metabolites from RB5. As a design, the evaluation of acute toxicity was performed. The bioluminescent test is broadly used to evaluate the potentially harmful effects of effluents discharged into surface waters (Griffitt et al., 2008). Hence, a Microtox assay was performed to evaluate the acute toxicity of the decolorization intermediates of RB5 by *S. halophilus* SSA-1575 under a high salt concentration. As depicted in Figure 11, the IR values of 25 and 50 mg/L RB5 (target dye) were 69 and 85%, respectively against *V. fischeri* after 30 min and confirmed high toxicity of this azo dye. After dye decolorization, the extracted metabolites were also evaluated for their acute toxicities. Clearly, the IRs of the extracted metabolites were significantly decreased (*p* < 0.001) to 7% (25 mg/L RB5) and 9% (50 mg/L RB5), respectively. Therefore, RB5 was significantly deconstructed by *S. halophilus* SSA-1575, into some non- toxic products rather than keeping its original molecule structure that may possess relatively high toxicity for carcinogenicity (Cury-Boaventura et al., 2004).

The biotransformation products and associated intermediates detected in our observations, such as *cis*-9-Octadecenoic, catechol and 3,7-dioxo-decahydronaphthalene-2,6-bis(olate), etc. indicate a microbial safety and its capability in a non-toxic decolorization processing on RB5 by *S. halophilus* SSA-1575. The *cis*-9-Octadecenoic was much less toxic to Jurkat cells when compared with linoleic acid (Cury-Boaventura et al., 2004). The 3,7-Dioxo-decahydronaphthalene-2,6-bis (olate), obtained from the biodegradation of Acid Scarlet 3R, was also confirmed to be a non-toxic compound (Kalyani et al., 2009). It has been reported that the catechol is a common intermediate compound in the aromatic degradation process, which can be converted into even less toxic aliphatic compounds before entering the TCA cycle (Cury-Boaventura et al., 2004). Based on the fact that aniline is a toxic aromatic amine used mainly in manufacturing dyes and dye intermediates, it was classified as B2, a probablehuman carcinogen (Cury-Boaventura et al., 2004). Additionally, aromatic solvents, such as benzene are common pollutants of the environment. Even at low concentrations, benzene is toxic to microorganisms and it can resist degradation at contaminated sites. However, the mechanism proposed by Gutiérrez et al. (1999) showed the efficiency of *Rhodococcus* sp. strain 33, to grow successfully in the presence of benzene and to use it as a precursor for synthesizing a saturated fatty acid (hexadecenoic acid, 16:1ω6c). Clearly, our findings in this safety evaluation, as well as a comparison with previous investigations, suggested that the yeast strain, *S. halophilus* SSA-1575 would potentially serve as a useful agent for detoxifying RB5, which can be safely implemented in a bioremediation process, particularly for those high-salt azo-dye wastewaters.

## Conclusion

This study displayed identification and characterization of a halotolerant yeast strain *S. halophilus* SSA-1575, capable of decolorizing and detoxifying the sulfonated diazo dye RB5 effectively under a static condition, which has been successfully identified from a WFT gut system. Clearly, the growing cells of *S. halophilus* SSA-1575 showed optimal growth and decolorization performance at 50 mg/L RB5, 5 g/L glucose, 0.08 g/L yeast extract, 0.05 g/L (NH_4_)_2_SO_4_, 30°C, pH 5 and in the presence of up to 50 g/L NaCl. UV-Vis spectroscopy and FTIR analyses of extracted products confirmed the biodegradation of RB5. NADH-DCIP reductase was identified as the key reductase, while Lac, LiP, and MnP were three oxidases which played a key role in the degradation and detoxification of RB5 by *S. halophilus* SSA-1575. A possible pathway of RB5 biodegradation was proposed based on Mass Spectrometry analysis and it seems to involve some key steps, including reductive decolorization, desulfonation, and deamination followed by mineralization through the TCA cycle. Furthermore, RB5 was obviously detoxified. The results improved the knowledge of azo dye-decolorizing *S. halophilus* species and provided a biological resource for bioremediation of industrial wastewater containing azo dyes and salts.

## Data Availability Statement

All datasets generated for this study are included in the article/supplementary material.

## Author Contributions

RA-T and SA designed the study and operated the experiments. RA-T wrote the original draft and formal analysis. SA analyzed and discussed the results, writing—review, and editing. E-RK analyzed chemical data. JS funding acquisition, validation, and writing—review and editing. All the authors agreed to be accountable for the content of the work.

## Conflict of Interest

The authors declare that the research was conducted in the absence of any commercial or financial relationships that could be construed as a potential conflict of interest.
